# AI Use in Psychotherapy Teaching and Training: The Perspective of Core Trainees in Coventry and Warwickshire Partnership Trust

**DOI:** 10.1192/bjo.2026.11345

**Published:** 2026-06-30

**Authors:** Uzair Asif, Alina Vaida

**Affiliations:** 1 CWPT, Coventry, United Kingdom; 2 CWPT, Warwick, United Kingdom

## Abstract

**Aims:**

To evaluate the impact of AI use on psychotherapy training for “core trainee” psychiatry residents. Psychotherapy training for residents includes theory-based teaching, courses, Balint groups, and completion of two psychotherapy cases across different modalities, all supervised by experienced psychotherapists. This includes anonymised case summaries, including a formulation.

**Methods:**

A short questionnaire about AI use was sent to core training psychiatry residents working in CWPT via email, asking about their use of AI in psychotherapy training. The data is both quantitative and qualitative, summarising emerging themes. A similar survey was done for England-based psychotherapy tutors and is reported in a separate poster.

**Results:**

14 out of 32 residents completed the questionnaire. 85.7% of responders reported they don’t have experience of using AI in psychotherapy training or teaching. Those who used it reported using it for questions about psychotherapy, advice on how to discuss certain topics, and to “consolidate summaries and formulations” or “to write up sessions”.

When combining the “yes” and “maybe” responses, most residents supported using AI for teaching psychotherapy concepts, assisting with scribing, and producing formulations (57.14%, 71.42%, and 71.42%, respectively). 28.57% did not support the use of AI-assisted formulation.

28.57% of residents expressed concerns about confidentiality, 35.71% mentioned concerns about a “lack of contextual or emotional understanding” by AI. 14.28% had concerns about AI replacing therapists in the future or about inputting data into AI models, which could facilitate this. 14.28% mentioned that the responses generated can be inaccurate.

When asked about the “best use” of AI 42.85% listed explaining or breaking down concepts (teaching). 14.28% suggested its use for scribing.

**Conclusion:**

Most respondents didn’t use AI in psychotherapy training or teaching. There is however significant support for its use for teaching, scribing and formulations. The residents expressed relevant concerns about the data protection and about AI lacking an in-depth understanding about emotions and context if being used in patient care.

A significant part of psychotherapy learning involves reflections about the sessions, facilitated by writing session summaries and supervision. The case formulation is a key part of the shared understanding, between resident, patient and the supervisor. AI being used to replace the process can negatively impact learning. Agreed guidance on best practice for the use of AI in psychotherapy training could assist residents in learning psychotherapy concepts and understanding potential pitfalls with AI use.

